# Autonomic Function Impairment and Brain Perfusion Deficit in Parkinson’s Disease

**DOI:** 10.3389/fneur.2017.00246

**Published:** 2017-06-08

**Authors:** Wei-Che Lin, Pei-Chin Chen, Chih-Cheng Huang, Nai-Wen Tsai, Hsiu-Ling Chen, Hung-Chen Wang, Kun-Hsien Chou, Meng-Hsiang Chen, Yi-Wen Chen, Cheng-Hsien Lu

**Affiliations:** ^1^Department of Diagnostic Radiology, Kaohsiung Chang Gung Memorial Hospital, Chang Gung University College of Medicine, Kaohsiung, Taiwan; ^2^Department of Neurology, Kaohsiung Chang Gung Memorial Hospital, Chang Gung University College of Medicine, Kaohsiung, Taiwan; ^3^Department of Biomedical Imaging and Radiological Sciences, National Yang-Ming University, Taipei, Taiwan; ^4^Department of Neurosurgery, Kaohsiung Chang Gung Memorial Hospital, Chang Gung University College of Medicine, Kaohsiung, Taiwan; ^5^Brain Research Center, National Yang-Ming University, Taipei, Taiwan

**Keywords:** autonomic function, arterial spin labeling, cortical perfusion, dopamine, Parkinson’s disease

## Abstract

**Introduction:**

Autonomic disorders have been recognized as important Parkinson’s disease (PD) components. Some vulnerable structures are related to the central autonomic network and have also been linked to autonomic function alterations. The aims of the study are to evaluate the severity of the autonomic dysfunction and the cortical hypoperfusion using arterial spin labeling (ASL) MRI. And then, possible relationships of significant between-group differences in perfusion pattern to clinical variables and autonomic functions were examined to determine the pharmaceutical effects of dopaminergic treatment on cerebral blood flow (CBF) in patients with PD.

**Methods:**

Brain ASL MRI was carried out in 20 patients with PD (6 men and 14 women, mean age: 63.3 ± 6.4 years) and 22 sex- and age-matched healthy volunteers to assess whole-brain CBF and the effects of dopaminergic therapy on perfusion. All subjects underwent a standardized evaluation of cardiovagal and adrenergic function including a deep breathing, Valsalva maneuver, and 5-min head-up tilt test. Perfusion MRI data were acquired on a 3.0 T scanner with a pulsed continuous ASL technique. The CBF, autonomic parameters, and clinical data were analyzed after adjusting for age and sex.

**Results:**

Patients exhibited a decline in autonomic function (rapid heart rate in response to deep breathing, low baroreflex sensitivity, high systolic and diastolic pressure, and altered tilting test response), widespread low CBF, and robust response to dopaminergic therapy. Lower perfusion in the middle frontal gyrus was associated with increased clinical disease severity (Unified Parkinson’s Disease Rating Scale I score, *P* < 0.001). Lower perfusion in autonomic control areas, such as the frontal lobe and insula, were significantly associated with autonomic impairment (*P* < 0.001).

**Conclusions:**

Our study indicates that PD is a progressive neurodegenerative disorder that changes the perfusion of central nervous system and is associated with variable autonomic dysfunctions. Neuronal loss and sympathetic activation may explain the interaction between cortical autonomic region perfusion and cardiovascular autonomic function.

## Introduction

Recently, autonomic disorders have been recognized as important Parkinson’s disease (PD) components, with considerable impact on health and quality of life (1–3). Previous studies demonstrate impairment of various autonomic domains, including sudomotor, cardiovagal, and adrenergic functions, in patients with PD (3). The autonomic nervous system contains several components and there seems to be dysregulation of more than one component of the autonomic nervous system in PD. The accumulation of compelling evidence for failure or dysregulation of sympathetic noradrenergic innervation of the cardiovascular system has been seen in PD (4). It is reasonable to suggest that systemic pathophysiology of PD is widespread, involves both the central and peripheral nervous systems, and is associated with autonomic dysfunction (5).

Neuroimaging studies have also shown extensive striatum and extra-striatal pathology (6), which has been linked to non-motor symptoms in PD (7). Cardiac I-123 metaiodobenzylguanidine (MIBG) imaging has been investigated in several neurodegenerative disorders associated with parkinsonism. Previous reports revealed that cardiac MIBG is significantly reduced in patients with PD (8). The delayed uptake of myocardial MIBG may reflect the functional status or tone of the sympathetic nervous system. Some vulnerable structures are related to the central autonomic network (CAN) and have also been linked to autonomic function alterations (9). The possible connection between autonomic dysfunction in PD and regional brain stem atrophy has been proposed in a previous study (10). However, the relationship between various domains of the autonomic dysfunction and the extensive change in central brain structure or cerebral perfusion is still unknown.

Perfusion abnormalities are potentially PD biomarkers that can be used to assess disease diagnosis, progression, and treatment response (11–13). Arterial spin labeling (ASL) is a non-invasive MRI perfusion tool employing arterial water as a freely diffusible endogenous tracer or contrast medium to quantitatively measure cerebral blood flow (CBF) per unit of tissue mass (14). The ability to quantify on an absolute scale further allows for assessment of cortical perfusion before and after a given intervention (13). Recent studies describe the clinical application of this technique to CBF quantification in PD (12, 13, 15). However, the relationship between autonomic dysfunction and cerebral perfusion alterations related to PD has not been critically examined.

Levodopa is the most important first-line drug for the management of PD, and it is almost always given in combination with the drug carbidopa. Levodopa treatment of PD can affect reflexive cardiovagal function (16), and decreased reflexive cardiovagal gain is more prominent in patients with long-standing PD (17), who would be expected to be on levodopa. Levodopa might significantly lower BP and HR at rest (18) as well as cutaneous vasodilation. In patients with orthostatic hypotension, levodopa might even worsen autonomic dysregulation (18). However, there is no conclusion about the relationships among autonomic function, brain perfusion, and dopaminergic therapy in PD to demonstrate the pharmaceutical effects of dopaminergic treatment.

We hypothesized a relationship between autonomic function and cortical perfusion alterations in PD. To test this hypothesis, ASL was used to measure cortical hypoperfusion in patients with PD before and after levodopa administration. Then, various domains of the autonomic nervous system were evaluated to identify the pattern and severity of autonomic dysfunction. Finally, possible relationships of significant between-group differences in perfusion pattern to clinical variables and autonomic functions were examined.

## Materials and Methods

### Participants and Study Design

The study protocol was approved by the local Research Committee. All participants or their guardians provided written informed consent prior to participation in the study. Twenty right-handed PD (6 men and 14 women, mean age: 63.3 ± 6.4 years) were prospectively enrolled in the Neurology Department. Patients were included if they had a definitive diagnosis of idiopathic PD (19) and had been followed up at the outpatient clinic for more than 6 months after titration of their daily dopaminergic medication to achieve an effective steady-state concentration.

Mean disease duration of PD was 2.48 ± 1.53 years. For comparison, 22 sex- and age-matched normal controls (NCs) (7 men and 15 women, mean age: 59.9 ± 6.0 years) with no medical history of neurologic diseases or psychiatric illnesses, alcohol/substance abuse, or head injury, and with similar levels of education, were recruited. These control participants underwent a single testing session without administration of any medication.

All evaluations, including evaluations of clinical disease status, autonomic function, MRI studies, and the Mini–Mental State Examination (MMSE) test, were initially assessed in the OFF state achieved by withdrawal of dopaminergic medications 12–18 h prior to testing. All patients underwent another MRI study for ASL evaluation 1 h after the patient had taken their daily dopaminergic medication. The daily dose of dopaminergic medication was converted into the equivalent dose of levodopa (20).

### Clinical Assessment

The disease severity and functional status of each patient were evaluated, as in our previous study (21), using the Unified Parkinson’s Disease Rating Scale (UPDRS), modified Hoehn and Yahr stage (HY-stage), and Schwab and England Activities of Daily Living Scale (SE-ADL).

### Evaluation of Cardiovascular Autonomic Function

All subjects underwent a standardized evaluation of cardiovagal and adrenergic function (22) including a deep breathing, Valsalva maneuver (VM), and 5-min head-up tilt (HUT) test according to previous report (23). Between the VM and HUT tests, a 5-min recording of the resting electrocardiogram and continuous blood pressure (BP) was taken to calculate spontaneous baroreflex sensitivity (BRS) and spectral analysis was used to measure heart rate variability (HRV).

The HR response to deep breathing (HR_DB) and Valsalva ratio were obtained through the appropriate tests. In addition, the maximal differences in systolic and diastolic blood pressure (BP) between baseline and after a 5-min HUT test were recorded as “SP difference” and “DP difference,” respectively.

Spontaneous BRS was computed using the sequence method (24), the Nevrokard BRS analysis program version 6.2.0 (Nevrokard Kiauta, Izola, Slovenia), and the following criteria: (1) systolic BP changes >1 mmHg; (2) sequences longer than 3 beats; and (3) correlation coefficient >0.85.

The beat-to-beat R-R interval power (in square millisecond) was reported for the high frequency (HF, 0.15–0.4 Hz) and low frequency (LF, 0.04–0.15 Hz) bands. The normalized low- and high-frequency power (LF normalized units, HF normalized units) were calculated as a percentage of overall power. The above computation was done by Matlab 7.1 (The Mathworks, Natick, MA, USA).

### Evaluation of Cerebral Perfusion

#### MR Image Acquisition

MR image acquisition and processing were as previously reported (21). High resolution T1-weighted imaging was performed on a 3.0 T scanner (GE Signa MRI, Milwaukee, WI, USA) with an eight-channel head coil. Perfusion MRI was performed on a 1.5 T scanner (Discovery MR450, GE Healthcare, Milwaukee, WI, USA) with an eight-channel head coil. ASL images were acquired using a pulsed continuous arterial spin labeling technique (13). The imaging parameters were: TR = 4,548 ms, postlabel delay = 1,525 ms, TE = 10.5 ms, matrix size = 128 × 128, number of excitations = 3, number of slices = 38, slice thickness = 4.0 mm (with whole-brain coverage), and total acquisition time = 4 min. ASL perfusion imaging sequence acquires two images: one shortly after the inflowing arterial spins are inverted (labeled image) and one without inverting the arterial spins (unlabeled image). Subtracting the labeled image from the unlabeled image produces an ASL perfusion weighted image, which can be converted to a quantitative image that reflects CBF. For each subject, a CBF map was calculated by a scanner console with FuncTool 3DASL (GE Healthcare) within 1 min, and CBF was reported in ml/100 g/min units.

#### Image Data Processing

Imaging data were preprocessed using FSL v5.0 (Functional Magnetic Resonance Imaging of the Brain Software Library^1^) and SPM8 (Statistical Parametric Mapping, Wellcome Department of Imaging Neuroscience, London, UK; available online at http://www.fil.ion.ucl.ac.uk/spm) implemented in Matlab 7.3 (MathWorks, Natick, MA, USA). All the image processing procedure, including tissue segmentation, study specific template construction, spatial normalization, and CBF partial volume effect (PVE) correction were conducted according to previous study (13) and was summarized in Supplementary Material.

### Statistical Analysis

#### Analysis of Demographic Data

The demographic data with continuous variables, including age, body mass index (BMI), and education level, were compared among the study groups using the two sample *t*-test for parametric data and the Mann–Whitney *U* test for non-parametric data, where appropriate, and are reported as the mean ± SD. The sex data were compared using the Pearson chi-square test. The autonomic parameters were compared using analysis of covariance (ANCOVA) and Mann–Whitney *U* test, where appropriate. The MMSE and TIV were analyzed using the ANCOVA model with the participant’s age, sex, and education level as covariates. The global tissue volumes, including the GM, WM, and CSF volumes, and global brain mean CBF were analyzed using the ANCOVA model with the participant’s age, sex, education level, and TIV as covariates. The associations between the clinical evaluations and the CBF values derived from the differences found *via* group comparisons were calculated by partial correlation analysis. The threshold for all statistical significance was set at *P* < 0.05.

#### Analysis of Regional CBF Differences between Groups

##### Comparisons between NC and Patients in the ON and OFF States

The differences in CBF maps were compared between the following groups: (1) NC vs. PD_OFF_ and (2) NC vs. PD_ON_. A voxel-wise ANCOVA design was used with age and sex as covariates. The command-line tool, AlphaSim, which is available in the AFNI toolbox (Analysis of Functional NeuroImages^2^), was used to correct the problem of image-based multiple comparisons. The statistical threshold for each voxel was set at a corrected *P*_alpha_ of 0.05 (whereas *P*_uncorrected_ was 0.005 with a cluster size of at least 155 voxels), based on the results of a Monte Carlo simulation.

##### Comparison between Patients in the ON and OFF States

The one-sample *t* test with levodopa equivalent dose as the covariate was used for voxel-wise comparisons between patients in PD_OFF_ vs. PD_ON_ states (*P*_alpha_ < 0.05).

#### Relationship between CBF, Autonomic Function, and Clinical Assessments

The mean CBF of the clusters with significant differences between NC and PD_OFF_, between NC and PD_ON_, and between OFF and ON status in the PD groups was extracted for further correlation analyses. Partial correlation analyses with age, sex, and BMI as nuisance covariates were performed to correlate the CBF differences and autonomic function parameters. A further analysis controlling for the levodopa equivalent dose was carried out to evaluate the relationship between interval changes of CBF values and the autonomic function parameters.

Similar analyses were also conducted to evaluate the relationship between CBF and clinical disease severity, and between autonomic parameters and clinical disease severity. The threshold for statistical significance was set at *P* < 0.05, with Bonferroni’s correction for multiple comparisons. All statistical analyses were performed by SPSS software (SPSS V.17, Chicago, IL, USA).

## Results

### Demographic Data

The demographic data are summarized in Table 1. Using ANCOVA analysis, it was determined that gray matter volume (*P* = 0.013) and CSF volume (*P* = 0.009) differed significantly among the NC and PD groups. The UPDRS total score, HY-stage, and SE-SAL in the OFF state were 33.9 ± 22.8, 2.0 ± 0.8, and 76.6 ± 30.0, respectively.

**Table 1:** Comparison of demographic, clinical, autonomic variables, and global anatomical measurements between the healthy controls and PD patients.

Variable	Control group	PD group	*P* value
	(*n* = 22)	(*n* = 20)	
Age (years)	59.9 ± 6.0	63.3 ± 6.4	0.187^a^
Sex (male/female)	7/15	6/14	0.899^b^
BMI	23.4 ± 3.1	23.8 ± 3.4	0.683^a^
Education (years)	11.5 ± 4.9	8.8 ± 4.4	0.053^e^
MMSE	27.1 ± 2.1	22.6 ± 7.4	0.092^c^
Systolic BP (mmHg)	128.5 ± 26.3	137.6 ± 28.6	0.139^e^
Diastolic BP (mmHg)	74.8 ± 12.0	78.3 ± 13.3	0.442^a^
Disease duration (years)		2.48 ± 1.53	
Levodopa equivalent dose (mg) (IQR)		214.0 ± 155.0	
UPDRS I-OFF		3.5 ± 3.0	
UPDRS II-OFF		7.9 ± 5.7	
UPDRS III-OFF		22.9 ± 15.1	
UPDRS total score-OFF		33.9 ± 22.8	
Modified HY-stage-OFF		2.0 ± 0.8	
SE-ADL-OFF		76.6 ± 30.0	
**Global anatomical measurements**			
GMV (L)	0.56 ± 0.04	0.52 ± 0.07	0.013^d^*
WMV (L)	0.55 ± 0.06	0.54 ± 0.07	0.832^d^
CSFV (L)	0.22 ± 0.03	0.24 ± 0.05	0.009^d^*
TIV (L)	1.32 ± 0.12	1.29 ± 0.15	0.937^c^
Global brain mean CBF-OFF (ml/100 g/min)	42.45 ± 6.4	36.83 ± 5.89	0.056^d^
Global brain mean CBF-ON (ml/100 g/min)	–	35.77 ± 5.72	0.012^d^*

*CBF, cerebral blood flow; CSFV, cerebrospinal fluid volume; BMI, body mass index; GMV, gray matter volume; IQR, interquartile range; MMSE, Mini–Mental State Examination; Modified HY-stage, modified Hoehn and Yahr stage; PD, Parkinson’s disease; SE-ADL, Schwab and England Activities of Daily Living Scale; TIV, total intracranial volume; UPDRS, Unified Parkinson’s Disease Rating Scale; WMV, white matter volume*.

*^a^Two sample t-test*.

*^b^Chi-square test*.

*^c^Analysis of covariance (ANCOVA) with adjustment for age, sex, and education*.

*^d^ANCOVA with adjustment for age, sex, education, and TIV*.

*^e^Mann–Whitney U test*.

*P value < 0.05.

### Autonomic Function Impairment in PD Patients

**Figure 1:**
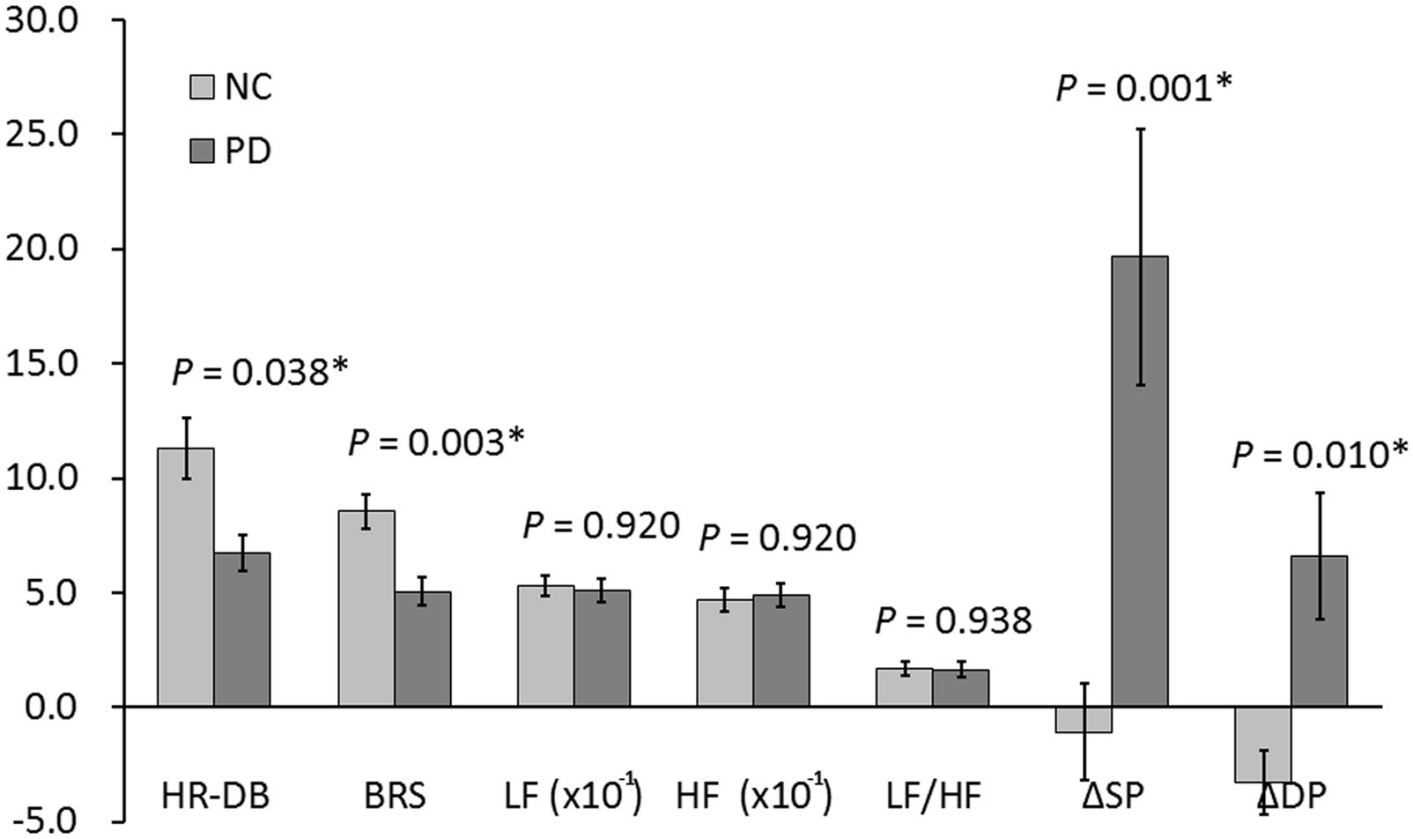
Autonomic functions in PD and NC. *P value < 0.05. BRS, baroreflex sensitivity; DP, diastolic pressure; HF, high frequency; HR_DB, heart rate response to deep breathing; LF, low frequency; NC, normal control; PD, Parkinson’s disease; SP, systolic pressure.

Analysis of covariance analysis revealed a significant difference in HR-DB, BRS, and tilt test-induced systolic and diastolic pressure changes between the NC and PD_OFF_ groups (*P* = 0.038, 0.003, 0.001, and 0.010, respectively) but not in frequency domain indices (Figure 1).

### Perfusion Deficits in PD Patients

#### CBF Comparisons between NCs and Patients in the ON and OFF States

##### NC vs. PD_OFF_

**Figure 2:**
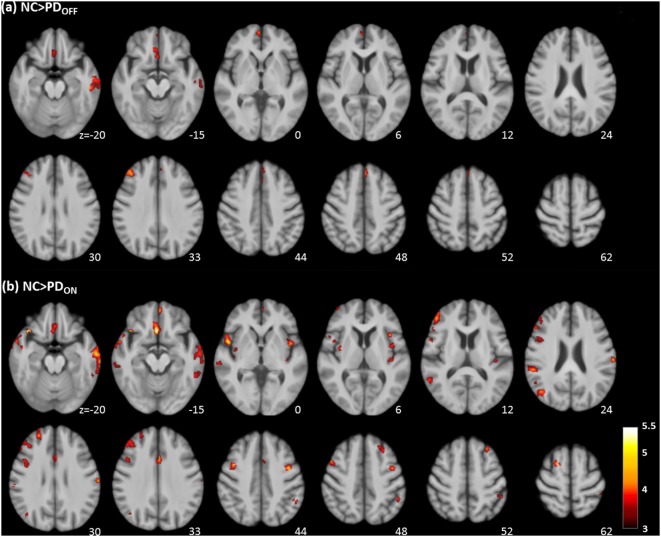
Regions with significantly decreased absolute cerebral blood flow in Parkinson’s disease (PD) patients in the OFF **(A)** and ON **(B)** states compared to healthy controls (cluster level statistics, *P* < 0.05, family-wise error corrected). The color bar indicates the T score.

Voxel-wise analysis of the absolute CBF maps revealed significant perfusion deficits in the frontal lobe, temporal lobe, and part of the limbic system of the PD_OFF_ group (Table 2; Figure 2A).

**Table 2:** Comparisons of cerebral blood flow levels in different brain regions between NCs and patients in the ON or OFF states.

MNI atlas coordinates			Voxel size	Region	*t*_max_
*X*	*Y*	*Z*			
**NC > PD_OFF_ (*P*_alpha_ < 0.05; *P*_uncorrected_ was 0.001 with a cluster size >155 voxels)**					
40	35	33	211	R middle frontal gyrus (BA 9)	4.52
–51	–24	–20	638	L inferior temporal gyrus (BA 20)	4.37
–58	–12	–20	–	L middle temporal gyrus (BA 21)	4.07
3	57	0	198	R medial frontal gyrus (BA 10)	4.34
–2	36	48	214	L superior frontal gyrus (BA 8)	4.29
2	21	–17	236	R subcallosal gyrus (BA 25)	4.23
8	32	–15	–	R anterior cingulate (BA 24)	4.04
**NC > PD_ON_ (*P*_alpha_ < 0.05; *P*_uncorrected_ was 0.001 with a cluster size >155 voxels)**					
3	22	–15	408	R anterior cingulate (BA 25)	5.76
39	22	–20	190	R inferior frontal gyrus (BA 47)	5.56
50	8	–2	720	R insula (BA 13)	5.34
57	6	–17	–	R superior temporal gyrus (BA 38)	4.78
–2	52	–14	171	L anterior cingulate (BA 32)	5.17
–3	58	–3	–	L medial frontal gyrus (BA 10)	4.42
–63	–19	28	189	L inferior parietal lobe (BA 40)	4.87
–57	–31	18	–	L superior temporal gyrus (BA 42)	3.43
–2	14	34	232	L cingulate gyrus (BA 24)	4.85
–62	–12	–21	2,104	L inferior temporal gyrus (BA 21)	4.83
–66	–22	–20	–	L inferior temporal gyrus (BA 20)	4.64
–68	–25	–9	–	L middle temporal gyrus (BA 21)	4.57
–40	0	45	241	L precentral gyrus (BA 6)	4.74
46	39	10	867	R middle frontal gyrus (BA 46)	4.64
44	48	13	–	R middle frontal gyrus (BA 10)	4.42
21	6	61	171	R middle frontal gyrus (BA 6)	4.54
–38	17	6	325	L insula (BA 13)	4.54
24	48	30	173	R superior frontal gyrus (BA 9)	4.49
56	–34	24	267	R insula (BA 13)	4.48
60	–24	31	–	R inferior parietal lobe (BA 40)	3.72
40	5	43	223	R middle frontal gyrus (BA 6)	4.48
44	–57	24	298	R middle temporal gyrus (BA 39)	4.43
–28	29	52	187	L middle frontal gyrus (BA 6)	4.27
–36	–21	4	159	L claustrum	4.21
–45	–12	4	–	L insula (BA 13)	3.63
–50	–42	52	223	L inferior parietal lobe (BA40)	4.13
62	–27	–6	391	R middle temporal gyrus (BA 21)	4.13
62	–25	3	–	R superior temporal gyrus (BA 22)	4.02
56	–30	–17	–	R inferior temporal gyrus (BA 20)	3.45
57	–49	12	218	R superior temporal gyrus (BA 22)	4.11
52	–54	22	–	R superior temporal gyrus (BA 39)	3.53
45	44	27	349	R superior frontal gyrus (BA 9)	4.07
46	14	34	–	R precentral gyrus (BA 9)	3.88
50	14	21	–	R inferior frontal gyrus (BA 9)	3.61
36	–3	6	181	R claustrum	4
**PD_OFF_ > PD_ON_ (*P*_uncorrected_ < 0.05, cluster size >100)**					
–38	–61	–50	353	L cerebellar tonsil	4.2
–30	11	57	215	L middle frontal gyrus (BA 6)	4.2
–9	–1	–18	131	L subcallosal gyrus (BA 34)	3.88

*BA, Brodmann area; MNI, Montreal Neurological Institute; NC, normal control; PD, Parkinson’s disease; R, right; L, left*.

##### NC vs. PD_ON_

Bilateral extensive perfusion decreases were found in the frontal, parietal, and temporal regions as well as the anterior cingulate, bilateral insula, and bilateral claustrum of the PD_ON_ group (Table 2; Figure 2B).

#### CBF Comparisons between the ON and OFF States in PD

**Figure 3:**
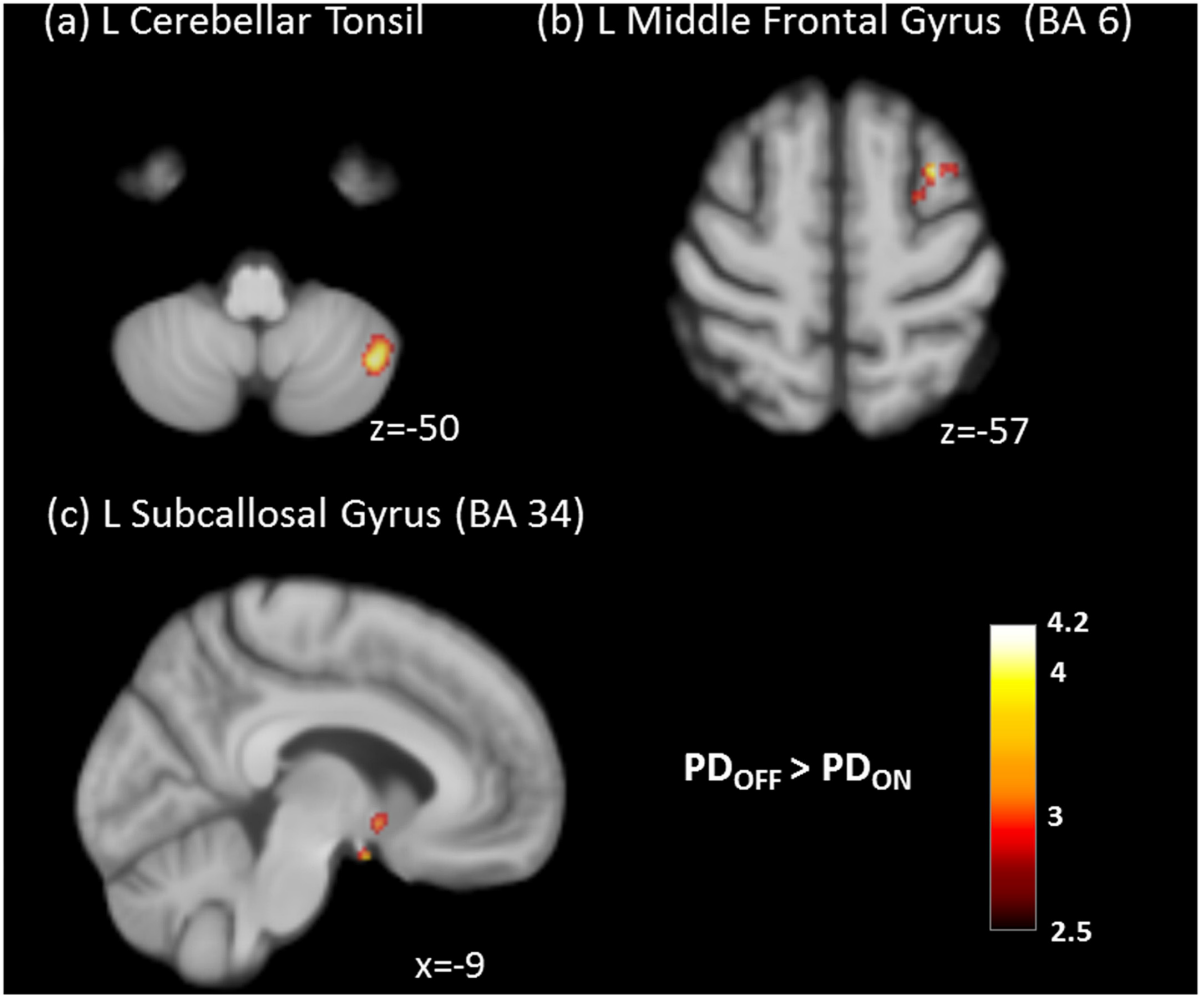
Significantly decreased absolute cerebral blood flow of **(A)** left cerebellar tonsil, **(B)** left middle frontal gyrus, and **(C)** left subcallosal gyrus in the ON state compared to the OFF state in Parkinson’s disease (PD) patients (cluster level statistics, *P* < 0.05, family-wise error corrected).

After the administration of dopaminergic medications, the PD group showed significantly decreased CBF in the cerebellum, frontal gyrus, and subcallosal gyrus (Table 2; Figure 3).

### Correlations between the CBF and All Clinical Assessment Results

#### Correlations with Disease Severity

Lower CBF in the middle frontal gyrus was associated with higher UPDRS I score (*P* < 0.001, *r* = –0.801) in the OFF state. Clinical severity assessment results were neither correlated with the CBF in the ON state nor with change in CBF from the OFF state to the ON state for the various brain regions.

#### Correlations with Autonomic Functions

Generally, lower CBF was associated with worse autonomic function (Table 3). In the OFF state, lower HR-DB was associated with lower CBF in medial frontal gyrus. Lower BRS was associated with lower CBF in the middle frontal gyrus, medial frontal gyrus, and superior frontal gyrus. Higher SP difference was associated with lower CBF in the medial frontal gyrus and superior frontal gyrus. Higher DP difference was associated with lower CBF in the superior frontal gyrus.

**Table 3:** Analysis of correlation between autonomic functions and group differences in CBF value after controlling for age, gender, and BMI.

	Autonomic functions (OFF state)		Anatomic location	*r*	*P*
OFF	HR-DB	R	Medial frontal gyrus	0.358	0.008
	BRS	R	Middle frontal gyrus	0.466	0.000
		R	Medial frontal gyrus	0.565	0.000
		L	Superior frontal gyrus	0.520	0.000
	SP difference in the tilting test	R	Medial frontal gyrus	–0.396	0.006
		L	Superior frontal gyrus	–0.472	0.001
	DP difference in the tilting test	L	Superior frontal gyrus	–0.402	0.006

ON	HR-DB	R	Inferior frontal gyrus	0.458	0.001
	BRS	R	Inferior frontal gyrus	0.448	0.001
		L	Insula	0.499	0.000
		R	Superior frontal gyrus	0.457	0.001

OFF-ON	BRS	L	Middle frontal gyrus	–0.679	0.008

*BMI, body mass index; BRS, baroreflex sensitivity; CBF, cerebral blood flow; DP, diastolic pressure; HR_DB, heart rate response to deep breathing; L, left; NC, normal control; R, right; SP, systolic pressure; UPDRS, Unified Parkinson’s Disease Rating Scale*.

In the ON state, lower HR-DB was associated with lower CBF in the inferior frontal gyrus. Lower BRS was associated with lower CBF in the inferior frontal gyrus, insula, and superior frontal gyrus.

Controlling for the levodopa equivalent dose, lower BRS was associated with smaller decrease in CBF values from the OFF state to the ON state in the middle inferior frontal gyrus.

### Correlations between the Autonomic Functions and Clinical Assessments

Lower SP difference was correlated with worsening measures of disease severity, including UPDRS II (*P* = 0.024, *r* = –0.561), UPDRS III (*P* = 0.020, *r* = –0.573), and UPDRS total score (*P* = 0.018, *r* = –0.582). Lower DP difference was also correlated with worsening measures of disease severity, including UPDRS I (*P* = 0.017, *r* = –0.587), UPDRS II (*P* = 0.001, *r* = –0.731), UPDRS III (*P* = 0.007, *r* = –0.647), and UPDRS total score (*P* = 0.003, *r* = –0.693) (Table 4).

**Table 4:** Correlation between autonomic function and disease severities.

Autonomic functions	Disease severity	*r*	*P*
SP difference in the tilting test	Unified Parkinson’s Disease Rating Scale (UPDRS) II	–0.561	0.024
	UPDRS III	–0.573	0.020
	UPDRS total	–0.582	0.018

DP difference in the tilting test	UPDRS I	–0.587	0.017
	UPDRS II	–0.731	0.001
	UPDRS III	–0.647	0.007
	UPDRS total	–0.693	0.003

## Discussion

Consistent with our hypothesis that the PD patients experienced autonomic dysfunction and cortical hypoperfusion as disease severity increased. Also, lower CBF in the OFF and ON states was associated with decline in autonomic functions. We demonstrated that the pathophysiology of PD involves both the central and peripheral nervous systems. Furthermore, our finding also support previous findings that the effects of dopaminergic therapies on cortical perfusion decline as the disease progresses. This study is the first to report associations among autonomic function, cortical hypoperfusion, and disease severity in PD and to highlight the clinical application of ASL in disease evaluation and therapeutic monitoring non-invasively.

In the present study, significant worsening of autonomic functions suggested the occurrence of both cardiovagal (parasympathetic) and adrenergic (sympathetic) autonomic dysfunctions in PD. These findings are in agreement with that multiple autonomic dysfunctions might occur in the early stage of PD (3). In addition. accumulating pathological changes in PD may explain the worsening of autonomic dysfunction (greater symptom burden with higher UPDRS score). However, it is still unknown and impossible to link the pathophysiology underlying this observation to adrenergic and cardiovagal autonomic function test results. The discrepancies between studies may be due to differences in sample size for each disease stage of PD and varying patient characteristics. For example, patients with advanced PD might be unable to stand for the tilting test due to severe postural instability.

Compared with the NCs, the PD patients also showed extensive cortical perfusion deficits, both in the ON and OFF states. The neuroimaging findings of past studies regarding cortical perfusion alterations have varied (11, 13). Our results and those of other studies showing widespread cortical hypoperfusion might be in accordance with studies showing: (i) early degeneration of various structures corresponding to different neuronal systems (25), (ii) decreased number of existing neurons resulting in decreased regional and global CBF (11), and (iii) altered functional connectivities previously shown to be associated with changes in regional/global neurovascular coupling, hemodynamic responses, and possibly earlier structural changes (26). A less likely argument is that cortical hypoperfusion is probably also explained by atrophy (27). However, by correcting for the PVE (28), we lessened the confounding influence of cortical atrophy in PD. Our results indicated that hypoperfusion might be involved in PD’s pathophysiology, but a single etiology is not sufficiently informative to completely explain it.

The patients with PD showed cortical hypoperfusion, particularly in the frontal lobe, temporal lobe, and salience network, such as the insula and anterior cingulate, in the ON and OFF states. Interestingly, areas of hypoperfusion highly overlap the telencephalic components of the CAN which are intimately connected with one another and with the hypothalamus and brain stem areas controlling autonomic function (9). Our results support the hypothesis that central autonomic dysfunction occurs even in the early stage of disease, which ascends caudo-rostrally from the lower brainstem through the forebrain into the cerebral cortex (25). Alteration of cortical perfusion could interfere with the CAN response to the heart rate input signaling in PD (29).

Frontal lobe hypoperfusion was associated with worse cardiovagal and adrenergic autonomic functions in the OFF state, while frontal lobe and insula hypoperfusions were associated with worse cardiovagal autonomic function in the ON state. Both animal and human studies support the crucial role of ventral medial prefrontal cortex activity in cardiovascular modulation. Inactivation of this region results in withdrawal of parasympathetic input to the baroreflex while maintaining sympathetic input (30). Functional MRI also revealed that during an isometric handgrip task, lower ventral medial prefrontal cortex activity correlates with higher heart rate and mean arterial pressure (31). Since sympathetic activity remained unchanged in their study, the hemodynamic changes were suggested to reflect vagal withdrawal. The ventral medial prefrontal cortex, insula, and amygdala–hippocampal complex are mainly involved in vagal modulation (32). The salience network (i.e., insula and cingulate) is the primary viscerosensory cortex and has a viscerotropic organization. The insula is also a visceromotor area, controlling both the sympathetic and parasympathetic outputs, primarily *via* a relay in the lateral hypothalamic area (33). Collectively, these autonomic dysfunctions and ASL deficits demonstrate again that both sympathetic and parasympathetic functions are affected in PD.

Perfusion was significantly decreased in the cerebellum, frontal lobe, subcallosal gyrus, and globus pallidus of patients in the ON state compared with the OFF state in the present study. Dopamine depletion is known to increase synchronous oscillatory activity in the corticostriatal networks (34), while levodopa can reduce functional connectivity between the ventral striatum, ventromedial prefrontal cortex, and cerebellum (35). Another study also suggested that dopaminergic medications and deep brain stimulation can shrink energetic demand due corticocortical synchronous oscillatory activity in PD and partly explain our finding of decreased cortical perfusion.

Our interpretation of the findings must be tempered by some study limitations. We did not perform autonomic function tests in the ON state. The effect of medication on autonomic function and its association with immediate changes in CBF before and after dopaminergic treatment needs to be determined. Second, this study investigated the relationship between perfusion of CANs and clinical autonomic impairment, but did not evaluate the peripheral autonomic system. The possible involvement of peripheral autonomic nerve system in PD will need the other researches to discuss. Furthermore, different dopaminergic medications might have different impacts on cortical perfusion, and their characteristic patterns in this study are unknown. We did not search for significant perfusion deficits in brainstem structures (hallmarks of autonomic dysfunction) since current ASL sequences are too inaccurate for brain stem evaluation. This topic awaits further investigation.

## Conclusion

We non-invasively revealed PD-related cortical blood flow via ASL. Our study indicates that PD is a progressive neurodegenerative disorder that changes the perfusion of central nervous system and is associated with variable autonomic dysfunctions. Because of its availability and repeatability, ASL might be helpful for disease monitoring and new therapeutic planning in the future.

## Ethics Statement

We declare that all human and animal studies have been approved by the Institutional Review Board of Chang Gung Memorial Hospital and have therefore been performed in accordance with the ethical standards laid down in the 1964 Declaration of Helsinki and its later amendments. We declare that all patients gave informed consent prior to inclusion in this study.

## Author Contributions

W-CL: research conception and organization, statistical analysis design, and manuscript writing. P-CC: execution of statistical analysis. C-CH: execution of research project. N-WT: execution of research project. H-LC: execution of statistical analysis. H-CW: execution of research project. K-HC: execution of statistical analysis. M-HC: execution of statistical analysis. Y-WC: execution of statistical analysis. C-HL: execution of research project, execution of research project, manuscript review, and critique.

## Conflict of Interest Statement

The authors declare that the research was conducted in the absence of any commercial or financial relationships that could be construed as a potential conflict of interest.

## Footnotes

^1^http://www.fmrib.ox.au.uk/fsl.

^2^http://afni.nimh.nih.gov/afni/.

## Abbreviations

PD, Parkinson’s disease; ASL, arterial spin labeling; CAN, central autonomic network; CBF, cerebral blood flow; UPDRS, Unified Parkinson’s Disease Rating Scale; HY-stage, Hoehn and Yahr stage; SE-ADL, Schwab and England Activities of Daily Living Scale; MMSE, Mini–Mental State Examination; NCs, normal controls; VM, Valsalva maneuver; HUT, head-up tilt; ECG, electrocardiogram; BRS, baroreflex sensitivity; HRV, heart rate variability; HR, heart rate; HR_DB, HR response to deep breathing; VR, Valsalva ratio; BP, blood pressure; SBP, systolic BP; RRI, R-R interval; pCASL, pulsed continuous arterial spin labeling; SPM, statistical parametric mapping; MNI, Montreal Neurological Institute; PVE, partial volume effect; ANCOVA, analysis of covariance; AFNI, Analysis of Functional NeuroImages.
