# Supplementary material for: Autonomic Function Impairment and Brain Perfusion Deficit in Parkinson’s Disease
Source: Front Neurol. 2017 Jun 8;8:246. doi: 10.3389/fneur.2017.00246 (PMC5462903; doi:10.3389/fneur.2017.00246)
Supplement: Supplementary file 1 [file Data_Sheet_1.DOC]

Image data processing

Imaging data were preprocessed using FSL v5.0 (Functional Magnetic Resonance Imaging of the Brain Software Library; http://www.fmrib.ox.au.uk/fsl) and SPM8 (Statistical Parametric Mapping, Wellcome Department of Imaging Neuroscience, London, UK; available online at http://www.fil.ion.ucl.ac.uk/spm) implemented in Matlab 7.3 (MathWorks, Natick, MA, USA). All T1 and ASL images for each participant were carefully checked by an experienced neuro-radiologist to ensure that they included no scanner artifacts, motion problems, or gross anatomic abnormalities.

To further ensure the accuracy of cross-modality image registration, the unlabeled ASL images and corresponding T1 images of each participant were skull-stripped using the Brain Extraction Tool v2.1 (BET and BET2; part of FSL) to remove non-brain tissues and background noise from the images [^1^](#_ENREF_1). For each participant, the brain mask that was generated from the unlabeled ASL images was also applied to the CBF map. We used a boundary-based registration algorithm implemented in FMRIB's Linear Image Registration Tool (FLIRT; part of FSL software) to obtain accurate and robust ASL and T1 image alignment [^2^](#_ENREF_2). To separate tissue types, the high resolution T1W image was used as the reference image to the extracted surfaces and then the unlabeled ASL image was aligned to the reference by maximizing the intensity gradient across tissue boundaries. Subsequently, the CBF maps were registered into the T1W space using transformation.

The DARTEL (Diffeomorphic Anatomical Registration Through Exponentiated Lie Algebra) [^3^](#_ENREF_3) toolbox implemented in SPM8 was used to generate group-specific templates for all the subjects based on their segmented GM and WM probability maps, and each group-specific template was registered into the Montreal Neurological Institute (MNI) standard space using affine transformation. Each individual subject's T1W image was mapped into the MNI space. With these two transformations, CBF maps in the T1W space were normalized to the group-speciﬁc template and mapped into the MNI space, and resliced to an isotropic voxel size of 1.5 mm. Because of the partial volume effect (PVE), CBF maps were corrected for volume atrophy according to the proportion of GM and WM in each voxel obtained from the segmented T1W image [^4^](#_ENREF_4). To eliminate outliers in the perfusion image due to large blood vessels or image processing computations, the threshold was set at a low value threshold of zero and a high value threshold of two standard deviations above the mean perfusion value for each subject [^5^](#_ENREF_5). Finally, the CBF maps after PVE correction were spatially smoothed using a 6-mm full-width at half-maximum Gaussian kernel for voxel-wise comparisons.

1. Smith SM. Fast robust automated brain extraction. Human brain mapping 2002;17(3):143-155.

2. Greve DN, Fischl B. Accurate and robust brain image alignment using boundary-based registration. Neuroimage 2009;48(1):63-72.

3. Ashburner J. A fast diffeomorphic image registration algorithm. Neuroimage 2007;38(1):95-113.

4. Johnson NA, Jahng GH, Weiner MW, et al. Pattern of cerebral hypoperfusion in Alzheimer disease and mild cognitive impairment measured with arterial spin-labeling MR imaging: initial experience. Radiology 2005;234(3):851-859.

5. Wang Z, Aguirre GK, Rao H, et al. Empirical optimization of ASL data analysis using an ASL data processing toolbox: ASLtbx. Magn Reson Imaging 2008;26(2):261-269.
